# Annual Recurrences of Viral Hemorrhagic Septicemia Epizootics in Age 0 Pacific Herring *Clupea pallasii* Valenciennes, 1847

**DOI:** 10.3390/ani11082426

**Published:** 2021-08-18

**Authors:** Paul K. Hershberger, Theodore R. Meyers, Jacob L. Gregg, Maya L. Groner, Sophie A. Hall, Hiruni T. Jayasekera, Ashley H. MacKenzie, Abigail S. Neat, Ella N. Piatt, Kyle A. Garver

**Affiliations:** 1 Marrowstone Marine Field Station, Western Fisheries Research Center, U.S. Geological Survey, Nordland, WA 98358, USA; jgregg@usgs.gov (J.L.G.); mgroner@usgs.gov (M.L.G.); sahall@usgs.gov (S.A.H.); hirunijayasekera97@gmail.com (H.T.J.); amackenzie@usgs.gov (A.H.M.); abbeysneat@gmail.com (A.S.N.); ellanpiatt@gmail.com (E.N.P.); 2 Juneau Fish Pathology Laboratory, Alaska Department of Fish & Game, P.O. Box 115526, Juneau, AK 99811, USA; ted.meyers@alaska.gov; 3 Prince William Sound Science Center, Cordova, AK 99574, USA; 4 Pacific Biological Station, Fisheries and Oceans Canada, Nanaimo, BC V9T 6N7, Canada; kyle.garver@dfo-mpo.gc.ca

**Keywords:** VHS, viral hemorrhagic septicemia, Pacific herring, epizootic

## Abstract

**Simple Summary:**

Pathogen surveillances in wild marine fish populations need to be carefully designed to address specific research or management objectives. Surveillance strategies should be designed around host life history characteristics, host and pathogen geographic ranges, laboratory diagnostic tools that are specific to the epidemiology of each pathogen, and the goal of the surveillance program. We demonstrate how the potential impacts of viral hemorrhagic septicemia can be under-appreciated in populations of Pacific herring by comparing results from opportunistically collected samples with those from more targeted epidemiological investigations that were focused on times and locations with high disease probability.

**Abstract:**

Throughout a 20 year biosurveillance period, viral hemorrhagic septicemia virus was isolated in low titers from only 6/7355 opportunistically sampled adult Pacific herring, reflecting the typical endemic phase of the disease when the virus persists covertly. However, more focused surveillance efforts identified the presence of disease hot spots occurring among juvenile life history stages from certain nearshore habitats. These outbreaks sometimes recurred annually in the same temporal and spatial patterns and were characterized by infection prevalence as high as 96%. Longitudinal sampling indicated that some epizootics were relatively transient, represented by positive samples on a single sampling date, and others were more protracted, with positive samples occurring throughout the first 10 weeks of the juvenile life history phase. These results indicate that viral hemorrhagic septicemia (VHS) epizootics in free-ranging Pacific herring *C. pallasii* are more common than previously appreciated; however, they are easily overlooked if biosurveillance efforts are not designed around times and locations with high disease potential.

## 1. Introduction

Viral hemorrhagic septicemia virus, genogroup IVa (hereafter referred to as VHS virus) has a broad host range in marine fishes throughout the coastal waters of North America and Asia, including China, Japan, and Korea [1,2,3]. Throughout parts of this range, the ecology of the virus is closely tied to Pacific herring (*Clupea pallasii* Valenciennes, 1847) and other hosts [4] which are highly susceptible to the resulting disease (viral hemorrhagic septicemia; VHS) and serve as effective reservoirs between outbreaks [5]. All available data indicate that apparently healthy hosts are responsible for virus perpetuation during these typical endemic periods via persistence in fully convalesced individuals [5]. Further, the reservoir of VHS virus in marine forage fishes such as Pacific herring *C. pallasii* and Pacific sardine represents a source of viral spillover to cultured Atlantic salmon in marine pet pens in Washington (USA) and British Columbia (Canada), which experience periodic VHS epizootics [3].

Epizootics of VHS periodically occur in wild marine fishes [1,2,3], presumably as a result of alignment between permissive environmental conditions and sympatry of host reservoirs and susceptible species. It is important to recognize that most epizootics go unnoticed because they remain small in scale and/or they occur in remote locations that are difficult to sample. Because of these logistical challenges, reports of VHS epizootics in wild marine fishes generally describe single sample collections from reported fish kills. As such, neither the temporal and spatial patterns nor the complete progression of natural VHS epizootics has been properly documented in wild marine fishes. Interestingly, although VHS virus occurs in Pacific herring *C. pallasii* from Puget Sound, Washington, USA, where the disease sometimes occurs in confined wild cohorts [6,7], epizootics among free-ranging herring have not yet been reported from this region [8].

This study was performed to document the interannual occurrence of VHS epizootics in Pacific herring *C. pallasii* from the same geographic locations in consecutive years and to document the progression of a single epizootic throughout the disease onset, peak, and recovery phases. To emphasize the ease with which these epizootics can be overlooked, we also report the results from 20 years of VHS virus surveillances in Pacific herring *C. pallasii* populations throughout the NE Pacific Ocean, including annual sampling from pre-spawn aggregations in Prince William Sound and Sitka Sound as well as opportunistic sampling from other locations.

## 2. Materials and Methods

Pacific herring *C. pallasii* (*n* = 7355) were collected from locations throughout coastal areas of the NE Pacific Ocean using various gear types and sampled for presence of VHS virus (Table 1). Spring samples (March–April) from Prince William Sound and Sitka Sound, AK consisted of pre-spawn or actively spawning fish that were collected in conjunction with state stock assessment surveys. Other collections were largely opportunistic, but some were expanded to targeted surveillances when possible. In accordance with requirements of the World Organization for Animal Health (OIE) for epidemiological surveillance, efforts were made to analyze 60 fish from each sample collection, as this sample size is appropriate for detecting an agent with 5% minimum expected prevalence. The VHS virus infection status was determined by plaque assay [9] of fresh or previously frozen (−80 °C unless noted) kidney/spleen pools on polyethylene glycol-pretreated [10] monolayers of *epithelioma papulosum cyprini cells* [11,12] in 24-well plates; the minimum detection threshold was 400 plaque forming units/g. Any samples demonstrating questionable cytopathic effects below this threshold were subjected to additional blind passage onto fresh EPC cells. VHS virus was confirmed in cell culture-positive samples using a TaqMan-based reverse transcription PCR (RT-qPCR) targeting the N-gene and sequencing to determine virus type and subtype [13].

**Figure 1:**
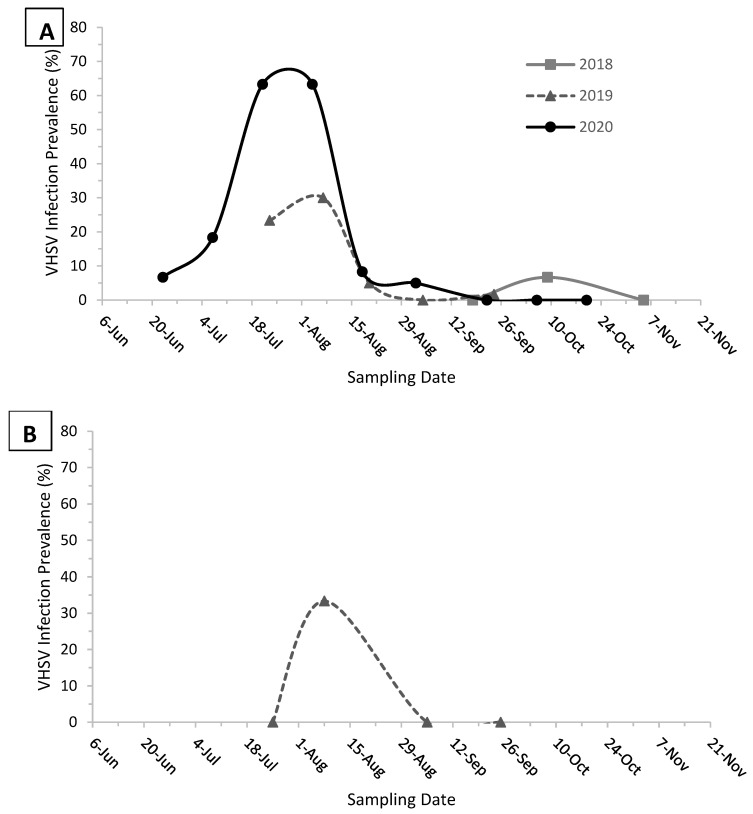
Progression of VHS virus epizootics in age 0 Pacific herring *C. pallasii* from the Puget Sound, WA region, including (**A**) Port Angeles Harbor (2018–2020) and (**B**) Port Ludlow Harbor (2019). Each data point reflects *n =* 30–60 fish.

More targeted investigations were launched in nearshore areas including embayments and boat harbors that were presumed hot spots for VHS (Table 2). Additionally, longitudinal sampling of juvenile Pacific herring *C. pallasii* from Port Angeles Harbor, WA, USA, occurred approximately every 2 weeks from 18 September to 5 November 2018, from 23 July to 24 September 2019, and from 23 June to 20 October 2020 (Figure 1A); analogous longitudinal sampling occurred from Port Ludlow Harbor, WA, USA, 25 July–25 September 2020. Samples were collected by cast net and consisted of 22–60 fish/day. All kidney/spleen samples were frozen at −70 °C (with the exception of 2014 samples from Puget Sound, which were frozen at −20 °C), then processed by viral plaque assay and viral isolates were confirmed as before.

## 3. Results

The typical endemic phase of VHS virus occurred during most sampling events in the period 2001–2020, when positive virus samples were detected in only 9/6277 Pacific herring *C. pallasii* from 8/117 independent sample collections (Table 1). During these endemic periods, viral titers in kidney/spleen pools from positive samples were at or below the minimum threshold of the plaque assay, and virus isolations could generally be confirmed only after blind passage onto fresh cells.

**Figure 2:**
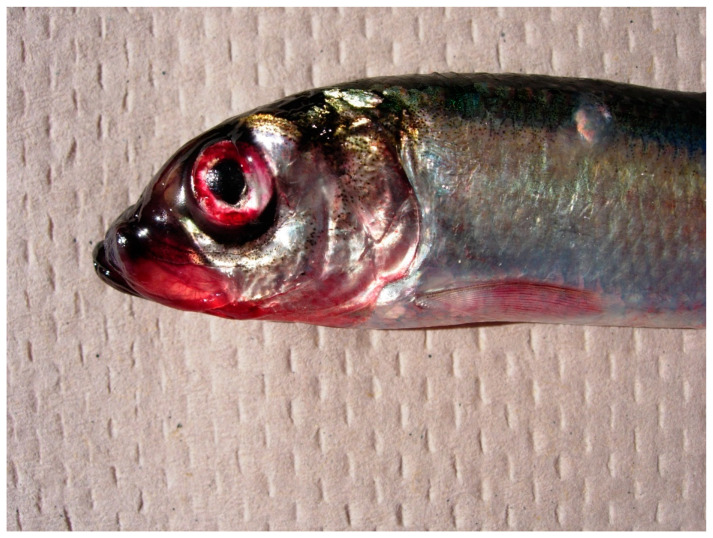
Pacific herring *C. pallasii* with gross external signs of VHS, including exopthalmia and hemorrhaging around the mouth, eyes, and fins.

Localized VHS epizootics in juvenile Pacific herring *C. pallasii* also occurred throughout the coastal areas of the NE Pacific Ocean, with some outbreaks occurring in the same locations in consecutive years (Table 2). An epizootic was detected in Sitka Sound, AK, USA (Bear Cove Bay) during March 2011 and was characterized by high infection prevalence (63%) and geometric mean tissue titer (6.8 × 10^3^ PFU/g) among positive samples. The fish were randomly sampled and dead/moribund individuals were not noted. This epizootic occurred at a time when cross sectional sampling indicated that adult conspecifics failed to test positive from Long Island, AK, USA, approximately 12 km away (Table 1). Another epizootic was detected in the San Juan Islands region of northern Puget Sound, WA during September 2014. Pacific herring *C. pallasii* with external signs of VHS, including focal and petechial hemorrhaging along the flank and fin bases were detected during beach seining sampling. Visual examinations estimated that lesions (Figure 2) occurred on approximately 10% of the fish during the peak of the outbreak. Samples reported in Table 2 were collected after the peak, when VHS virus was detected from 13 to 27% of the lesioned fish that were stored at −20 °C, a sub-optimal condition for virus isolation. A third epizootic was detected in Port Ludlow Harbor, Puget Sound, WA, USA, on 8 August 2019, when 33% of juveniles tested positive for VHSV. That outbreak was short lived, as samples from the same location failed to test positive either 14 d before or 28 d after this event (Figure 1B). Recurring epizootics occurred among newly-metamorphosed juveniles in Hot Spring Cove, BC, Canada (June 2018 and 2019) and Port Angeles Harbor, WA, USA (summer 2019 and 2020). Longitudinal sampling from the later site indicated that protracted epizootics occurred in 2019 and 2020. Sampling in 2018 commenced in September, after the epizootic would have been expected to subside based on data from the subsequent years (Figure 1A). Sampling in 2019 commenced on 23 July, just prior to the disease peak on 7 August, which was characterized by 30% infection prevalence and geometric mean viral tissue titers of 9.4 × 10^4^ PFU/g. Sampling in 2020 began even earlier, when larvae were metamorphosing to juveniles; infection prevalence increased from 6.7% on 23 June peaked at 63% from 21 July to 4 August, and declined to 0% by 22 September (Figure 1A); geometric mean viral tissue titers were 1.2 × 10^4^ PFU/g during the peak.

## 4. Discussion

Results from 20 years of surveillance efforts indicate that attempted VHS virus isolations from opportunistically collected adult Pacific herring *C. pallasii* largely return negative results (Table 1), and this traditional biosurveillance approach offers limited value for locating epizootics or informing disease management decisions. Despite these largely negative results, these apparently healthy individuals serve as a collective host reservoir by covertly perpetuating VHS virus [5], as outbreaks can often be stimulated in these individuals after their capture, transfer, and confinement [6,7]. Unfortunately, this traditional biosurveillance approach, involving attempted virus isolation, falls short of being able to deduce covert VHS virus carrier states, individual exposure histories, or population impacts of the disease. These results emphasize the need to consider the epidemiology of each disease and define the objectives of field surveillances before committing to survey design and laboratory diagnostics. For example, when attempting to incorporate the impacts of VHS into population assessment models, serological surveillances that deduce exposure histories may provide more informative than traditional virus isolation techniques. Additionally, recent investigations into VHS virus perpetuation mechanisms indicate that future efforts to deduce VHSV exposure histories in Pacific herring *C. pallasii* may be better served using alternative diagnostic techniques and tissues (i.e., RT qPCR on gill tissues) that provide a much more accurate view of exposure history than attempted virus isolation from kidney/spleen homogenates [5].

Unlike random or opportunistic surveillances in adult Pacific herring *C. pallasii*, which largely returned negative results, targeted surveillances in juvenile cohorts effectively identified periodic VHS epizootics, some of which recurred at the same time and locations in multiple years (Table 2). Some epizootics occurred over short durations and were detected only at a single timepoint in longitudinal assessments (e.g., Port Ludlow Bay in 2019), and others were more protracted, lasting several months (e.g., Port Angeles Harbor 2020; Figure 1). These differences in disease kinetics may reflect differences in site-specific difference in fish density and behavioral vagility. The host and environmental disease cofactors associated with these epizootics remain unexplored; however, several site-specific observations were common among all outbreaks. First, all the epizootics occurred among juvenile herring, and most (except for Sitka Sound in 2011, which involved age 1+ cohorts) occurred shortly after larval metamorphosis to the juvenile life stage. Second, all epizootics were detected in nearshore areas of embayments or boat harbors containing limited water exchange, where juvenile cohorts occurred in high abundances for extended periods. Finally, although mass mortalities were generally not observed in association with the epizootics (apart from Hot Springs Cove, 2018 and 2019), the outbreaks likely contributed to elevated mortality by predation, as birds and marine mammals were observed consuming distressed fish that were demonstrating abnormal behaviors such as whirling on their longitudinal axes. When combined, these commonalities suggest that some sites are more conducive to VHS outbreaks than others, and that small-scale VHS epizootics in Pacific herring *C. pallasii* occur more frequently in nearshore areas than previously appreciated. This observation is extended to geographical areas such as Puget Sound, WA, USA, where the virus was previously known to exist, but epizootics were never previously reported in free-ranging wild fish [8], presumably because prior VHS virus surveillances were not directed towards high probability locations and times. These documented singular and recurring epizootics in understudied nearshore habitats suggest that the population-level impacts of VHS to Pacific herring *C. pallasii* are likely to be underestimated until targeted wild fish surveillances are directed towards the specific times and geographic locations that are most permissive for the disease.

The demonstrated frequency of VHS epizootics among age 0 Pacific herring *C. pallasii* in nearshore habitats raises questions regarding the accuracy of host/pathogen coevolution paradigms in the context of a highly virulent pathogen like VHS virus. For example, in the “avirulence hypothesis”, virulence is assumed to be a consequence of the pathogen’s maladaptation to a new host and environment; as such, virulence should be expected to attenuate in long standing host/pathogen relationships [14]. However, VHS virus has likely occurred in the NE Pacific Ocean for several centuries [15], where it has a broad host range that includes a long-standing relationship with Pacific herring *C. pallasii*. Despite this long-term sympatry, epizootics continue to occur and adaptation towards avirulence or resistant host phenotypes has not occurred. Because there is no published evidence of cross-generational transfer of antibodies, annual outbreaks in age 0 cohorts likely occur due to the annual influx of naïve susceptible individuals into the population via larval metamorphosis. This example fails to support the “avirulence hypothesis” and provides support for the more contemporary “trade-off hypothesis” [16,17], whereby the pathogen cost for maintaining fitness through higher virulence can be out-weighed by certain benefits, such as increased transmission with shortened infection duration. This second hypothesis more appropriately explains the Pacific herring *C. pallasii*/VHS virus interactions, where virulence is maintained concomitantly with high shedding and transmission rates [18] and is analogous to the maintenance of infectious hematopoietic necrosis virus (IHNV) virulence in salmonids [19]. This perpetuation of VHS virus virulence in populations with long-standing host/pathogen co-evolution suggests that epizootics are likely to continue in many regions, including the NE Pacific Ocean, and in other geographic areas such as the Laurentian Great Lakes, where a different VHS virus lineage (Genotype IVb) recently emerged [20].

Shortly after their metamorphosis from larvae to juveniles, age 0 Pacific herring *C. pallasii* adopt a portfolio of life history strategies, whereby some remain in nearshore zones and others quickly venture into more open water pelagic habitats. An immediate reduction in survival potential may be incurred among those utilizing the former habitat, owing to decreased growth rates (unpublished data) and higher potential for VHS outbreaks compared to those subscribing to the open water phenotype. This immediate reduction in survival potential may be offset by long term immunological gains among the survivors, which generate adaptive resistance against future outbreaks of the disease [21]. Future research would be helpful to address this herd immunity hypothesis.

## Figures and Tables

**Table 1:** Voral hemorrhagic septicemia (VHSV) virus survey results from Pacific herring *C. pallasii* during typical endemic periods.

Year	Herring Stock	Collection Site	Collection Date	Gear Type	Adult/ Juvenile (A/J)	Mean Fork Length mm (SD)	VHSV Detection by Cell Culture (No. Positive/*n*)
2001	Cook Inlet, AK, USA	Kachemak Bay	18 June	Purse Seine	J	NA	0% (0/32)
2002	Cook Inlet, AK, USA	Kamishak Bay	20 May	Purse Seine	A	NA	0% (0/20)
2003	Puget Sound, WA, USA	Port Orchard	6 February	Trawl	A	179 (19)	0% (0/60)
		Port Madison	6 February	Trawl	A	173 (19)	0% (0/60)
		Skagit Bay	12 February	Trawl	A	161 (10)	0% (0/60)
		Port Susan	12 February	Trawl	A	171 (14)	0% (0/60)
		Port Gamble Bay	25 February	Trawl	J	122 (24)	0% (0/60)
2004	Puget Sound, WA, USA	Cherry Point	4 May	Trawl	A	221 (16)	1.7% (1/60)
	N. Hecate Strait, AK, USA	Behm Canal	11 April	Purse Seine	A	NA	0% (0/20)
	Frederick Sound, AK, USA	Hidden Falls	9 April	Cast Net	J	NA	0% (0/15)
2005	Cook Inlet, AK, USA	Dry Bay	16 May	Purse Seine	A	NA	0% (0/53)
2006	Cook Inlet, AK, USA	Nordyke Bay	18 May	Purse Seine	A	NA	0% (0/60)
		Oil Bay	21 May	Purse Seine	A	NA	0% (0/60)
		Iniskin Bay	22 May	Purse Seine	A	NA	0% (0/60)
2007	Prince William Sound, AK, USA	St. Matthews Bay	5 April	Cast Net	A	224 (17)	0% (0/60)
		Simpson Bay	19 April	Purse Seine	J	86 (6.3)	0% (0/60)
		Sawmill Bay	30 November	Purse Seine	A	215 (21)	0% (0/60)
		Simpson Bay	2 December	Purse Seine	A	187 (13)	0% (0/60)
	Sitka Sound, AK, USA	Cannon Island	9 April	Cast Net	A	215 (18)	0% (0/60)
	Kodiak, AK, USA	Uganik Bay	18 February	Purse Seine	A	NA	0% (0/30)
		Uyak Bay	21 February	Purse Seine	A	NA	0% (0/35)
	Cook Inlet, AK, USA	Iniskin Bay	16 May	Purse Seine	A	NA	1.7% (1/60)
		Amakdedulia Cove	27 May	Purse Seine	A	NA	1.7% (1/60)
		Ursus Cove	27 May	Purse Seine	A	NA	0% (0/60)
2008	Prince William Sound, AK, USA	Unknown	17 March	Purse Seine	J	141 (11)	0% (0/60)
		Fish Bay	19 March	Purse Seine	A	236 (27)	0% (0/45)
		Evans Point	24 March	Purse Seine	A	208 (18)	0% (0/60)
		Whale Bay	24 March	Purse Seine	J	149 (22)	0% (0/60)
		St. Matthews Bay	8 April	Purse Seine	A	NA	0% (0/32)
		Port Gravina	8–12 November	Purse Seine/Jig	A	197 (23)	0% (0/80)
	Sitka Sound, AK, USA	N. Middle Island	26 March	Purse Seine	A	249 (14)	0% (0/60)
2009	Prince William Sound, AK, USA	Port Gravina	20 March	Purse Seine	A	199 (15)	0% (0/60)
		Port Gravina	20 March	Purse Seine	A	168 (11)	0% (0/60)
		Simpson Bay	22 March	Purse Seine	J	94 (8)	0% (0/60)
		Eaglek Bay	14 November	Gill Net	J	98 (4)	0% (0/29)
		Port Gravina	15 November	Purse Seine	A	179 (17)	0% (0/60)
		Lwr. Herring Bay	16 November	Gill Net and Trawl	J	99 (4.0)	0% (0/14)
		Elrington Pass	17 November	Purse Seine	A	216 (19)	0% (0/60)
		Simpson Bay	19 November	Purse Seine	J	87 (14)	0% (0/60)
		Simpson Bay	19 November	Cast Net	J	70 (12)	0% (0/33)
	Sitka Sound, AK, USA	Unknown	24 March	Purse Seine	A	270 (19)	0% (0/44)
		St. John Babtist Bay	26 March	Purse Seine	A	248 (23)	0% (0/67)
		Unknown	27 March	Purse Seine	J	175 (7)	0% (0/68)
	Cook Inlet, AK, USA	Chenik Bay	8 May	Purse Seine	A	NA	0% (0/60)
		Rocky Cove	21 May	Purse Seine	A	NA	0% (0/59)
2010	Prince William Sound, AK, USA	Port Gravina	16 March	Purse Seine	A	213 (14)	0% (0/60)
		Port Fidalgo	19 March	Purse Seine	A	200 (15)	0% (0/60)
		Simpson Bay	20 March	Purse Seine	J	109 (23)	2–5% ^A^
		Cordova Harbor	2–13 June	Cast Net	J	85 (11)	0% (0/49)
		Cordova Harbor	18 August	Cast Net	J	35 (6.8)	0% (0/54)
		Cordova Harbor	28 September– 7 October	Cast Net	J	50 (5.7)	0% (0/22)
		Whale Bay	10–11 November	Gill Net	J	95 (33)	1.7% (1/60)
	Sitka Sound, AK, USA	Indian River	22 March	Purse Seine	A	242 (22)	0% (0/60)
		Kruzof Island	24 March	Purse Seine	A	241 (25)	0% (0/60)
2011	Prince William Sound, AK, USA	Lower Herring Bay	11 March	Cast Net	J	95 (3.9)	3.3% (2/60)
		Eaglek Bay	15 March	Cast Net	J	113 (22)	0% (0/60)
		Port Fidalgo	16 March	Cast Net	J	76 (5.7)	0% (0/60)
		St. Matthew’s Bay	2 April	Jig	A	246 (19)	0% (0/60)
		Port Gravina	4 April	Cast Net	A	219 (20)	0% (0/60)
		Port Fidalgo	6 April	Purse Seine	A	253 (12)	0% (0/60)
		Simpson Bay	13 October	Cast Net	J	52 (3.0)	0% (0/43)
		Simpson Bay	15 November	Cast Net	J	60 (6.1)	0% (0/60)
		Whale Bay	20 November	Cast Net	J	83 (6.4)	0% (0/60)
		Port Gravina	21 November	Purse Seine	A	205 (19)	0% (0/30)
		Port Gravina	22 November	Purse Seine	A	157 (11)	0% (0/30)
		Simpson Bay	13 December	Cast Net	J	60 (5.0)	0% (0/60)
	Sitka Sound, AK, USA	Long Island	22 March	Purse Seine	A	232 (16)	0% (0/60)
	Cook Inlet, AK, USA	Bruin Bay	4 May	Purse Seine	A	NA	0% (0/60)
		Rocky Cove	13 May	Purse Seine	A	224 (60) ^B^	0% (0/60)
	Bristol Bay, AK, USA	Togiak Bay	9 May	Purse Seine	A	NA	0% (0/60)
2012	Prince William Sound, AK, USA	Simpson Bay	11 January	Cast Net	J	57 (2.8)	0% (0/60)
		Glacier Island Pass	8 February	Dip Net	A	240 (13)	0% (0/15)
		Eaglek Bay	21 March	Gill Net	J	99 (3.2)	0% (0/30)
		Port Gravina	28 March	Purse Seine	A	218 (16)	0% (0/60)
		Port Gravina	31 March	Purse Seine	A	216 (16)	0% (0/60)
		Fidalgo Bay	2 April	Purse Seine	A	231 (20)	0% (0/60)
		Simpson Bay	20 April	Cast Net	J	78 (16)	0% (0/30)
		Port Gravina	9 November	Cast Net	J	63 (5.5)	0% (0/30)
		Simpson Bay	9 November	Cast Net	J	72 (9.0)	0% (0/30)
		Zaikoff Bay	13 November	Cast Net	J	72 (4.4)	0% (0/60)
		Lower Herring Bay	15 November	Cast Net	J	90 (4.3)	0% (0/30)
		Port Gravina	15 November	Purse Seine	A	159 (14)	0% (0/60)
	Sitka Sound, AK, USA	N. Kasiana Isl.	3 April	Cast Net	A	233 (22)	0% (0/60)
		St. John Bay	4 April	Purse Seine	A	214 (24)	0% (0/60)
		Sitka Harbor	4 April	Cast Net	A	225 (22)	0% (0/60)
	Cook Inlet, AK, USA	Bruin Bay	7 May	Purse Seine	A	254 (10) ^B^	0% (0/60)
2013	Prince William Sound, AK, USA	Port Gravina	27 March	Purse Seine	J	147 (16)	0% (0/60)
		Port Gravina	31 March	Purse Seine	A	232 (20)	0% (0/60)
		Port Gravina	1 April	Purse Seine	A	225 (23)	0% (0/60)
		Lower Herring Bay	9 November	Trawl, Cast Net	J	93 (9.7)	0% (0/60)
		Port Gravina	13 November	Cast Net	J	90 (6.0)	0% (0/40)
		Cordova Harbor	20 November	Cast Net	J	70 (7.5)	0% (0/60)
	Sitka Sound, AK, USA	Apple Islands	29 March	Cast Net	A	246 (28)	0% (0/60)
		Silver Bay	30 March	Cast Net	A	251 (16)	0% (0/60)
		Unknown	30 March	Cast Net	A	226 (26)	0% (0/60)
	Kodiak, AK, USA	Kiliuda Bay	24 April	Purse Seine	A	282 (60) ^B^	9.1% (1/11)
	Cook Inlet, AK, USA	Akumwarvik Bay	20 May	Purse Seine	A	219 (60) ^B^	0% (0/60)
2014	Prince William Sound, AK, USA	Sheep Bay	26 March	Purse Seine	A	216 (14)	1.7% (1/60)
		Port Fidalgo	28 March	Purse Seine	A	228 (15)	0% (0/60)
		Port Gravina	29 March	Purse Seine	A	242 (14)	0% (0/60)
		Simpson Bay	15–23 November	Trawl	J	78 (12)	0% (0/60)
		Port Gravina	16 November	Trawl	J	70 (5.1)	0% (0/61)
		Eaglek Bay	19 November	Trawl	J	96 (4.3)	0% (0/61)
	Cook Inlet, AK, USA	Kamishak Bay	30 April	Unknown	A	NA	0% (0/60)
		Kamishak Bay	13 May	Unknown	A	NA	0% (0/59)
	Sitka Sound, AK, USA	Sitka Harbor	26 March	Cast Net	A	245 (25)	0% (0/60)
		Middle Island	27 March	Cast Net	A	241 (30)	0% (0/60)
		Inner Point	28 March	Purse Seine	A	222 (20)	0% (0/60)
2015	Prince William Sound, AK, USA	Port Gravina	3 April	Cast Net	A	228 (17)	0% (0/60)
		Simpson Bay	6 November	Trawl	J	82 (11) ^B^	0% (0/46)
		Lower Herring Bay	11 November	Gill Net	J	85 (5.0)	0% (054)
		Whale Bay	12 November	Trawl	J	89 (7.2)	0% (0/60)
	Cook Inlet, AK, USA	Chenik Bay	27 April	Purse Seine	A	239 (10) ^B^	0% (0/60)
	Sitka Sound, AK, USA	Bieli Rock	20 March	Purse Seine	A	239 (26)	0% (0/60)
		Bieli Rock	22 March	Purse Seine	A	250 (22)	0% (0/60)
		Bieli Rock	22 March	Purse Seine	A	231 (24)	0% (0/60)
	Ketchican, AK, USA	Craig	17 December	Purse Seine	A	193 (18)	0% (0/76)
2016	Prince William Sound, AK, USA	Red Head Point	7 April	Jig	A	205 (17)	0% (0/60)
		Knowles Head	8 April	Cast Net	A	223 (24)	0% (0/120)
		Simpson Bay	29 October	Trawl	J	82 (3.5)	0% (0/60)
		Eaglek Bay	30 October	Trawl	J	95 (4.6)	0% (0/60)
		Lower Herring Bay	2 November	Trawl	J	96 (4.4)	0% (0/60)
	Sitka Sound, AK, USA	S. Salsbury Sound	21 March	Purse Seine	A	218 (22)	0% (0/60)
		North Crest	22 March	Purse Seine	A	215 (13)	0% (0/60)
		Point Brown	22 March	Cast Net	A	217 (24)	0% (0/60)
2017	Prince William Sound, AK, USA	Port Gravina	7 April	Purse Seine	A	195 (14)	0% (0/60)
		Rocky Bay	10 April	Purse Seine	J	140 (46)	0% (0/60)
		Port Fidalgo	10 April	Purse Seine	A	191 (14)	0% (0/60)
	Sitka Sound, AK, USA	Unknown	24 March	Cast Net	A	221 (14)	0% (0/60)
		S. Magoun Island	25 March	Cast Net	A	225 (15)	0% (0/60)
		Unknown	25 March	Cast Net	A	225 (16)	0% (0/60)
2018	Prince William Sound, AK, USA	Port Fidalgo	10–11 April	Purse Seine	J	152 (36)	0% (0/60)
		Cedar Bay	12 April	Purse Seine	A	201 (15)	0% (0/60)
		Rocky Bay	13 April	Purse Seine	A	204 (16)	0% (0/60)
	Sitka Sound, AK, USA	Guide Island	22 March	Cast Net	A	221 (15)	0% (0/56)
		Unknown	23 March	Cast Net	A	215 (16)	0% (0/49)
		Kruzof Island	23 March	Cast Net	A	224 (23)	0% (0/72)
2019	Puget Sound, WA, USA	Oak Bay	29 August	Dip Net	J	103 (3.7)	0% (0/60)
	Prince William Sound, AK, USA	Double Bay	5 April	Purse Seine	A	176 (14)	0% (0/60)
		Canoe Pass	6 April	Purse Seine	A	157 (29)	0% (0/60)
		Windy Bay	6 April	Purse Seine	A	179 (13)	0% (0/59)
	Sitka Sound, AK, USA	Krestof Isl.	25 March	Cast Net	A	205 (18)	0% (0/60)
		Krestof Isl.	26 March	Cast Net	A	197 (17)	0% (0/60)
		Whitestone Narrows	27 March	Cast Net	A	204 (18)	0% (0/60)
2020	Puget Sound	Eagle Harbor	26 August	Cast Net	J	69 (3.4)	0% (0/60)
	Prince William Sound, AK, USA	Canoe Pass	8 April	Purse Seine	A	213 (13)	0% (0/130)
		Double Bay	10 April	Purse Seine	A	220 (26)	0% (0/59)
	Sitka Sound, AK, USA	Kruzof	31 March	Cast Net	A	215 (14)	0% (0/60)
		Low Island	1 April	Cast Net	A	215 (14)	0% (0/60)
		Silver Bay	2 April	Cast Net	A	204 (10)	0% (0/60)
	Kodiak, AK, USA	Uganik Bay	21 April	Purse Seine	A	199 (10) ^B^	0% (0/60)

“NA” indicates data “Not Available.” ^A^ A single sample containing pooled tissues from 3 Pacific herring *C. pallasii* tested positive (*n =* 60) for VHSV after blind passage. Therefore, the prevalence was 1–3/60. ^B^ Fish lengths were recorded as standard length, rather than fork length.

**Table 2:** Viral hemorrhagic septicemia virus (VHSV) survey results from Pacific herring *C. pallasii* during epizootic periods.

Region	Year	Stock	Collection Site	Collection Date	Gear Type	Adult/Juvenile (A/J)	Mean Fork Length mm (SD)	VHSV Detection by Cell Culture (No. Positive/n)
AK, USA	2011	Sitka Sound	Bear Cove Bay	24 March	Cast Net	J	108 (11)	63% (38/60)
BC, Canada	2018	W. Vancouver Isl.	Hot Springs Cv.	24 June	Dip Net	J	NA ^B^	85% (22/26)
	2019	W. Vancouver Isl.	Hot Springs Cv.	27 June	Beach Seine	J	NA	96% (29/30)
WA, USA	2014	Puget Sound	Lopez Isl. ^A^	11 September	Beach Seine	J	NA ^C^	27% (6/22)
		Puget Sound	Waldron Isl. ^A^	12 September	Beach Seine	J	NA ^C^	13% (3/24)
	2018	Puget Sound	Pt. Angeles Hbr.	18 September–5 November	Cast Net	J	67 (8.0)–78 (7.4)	6.7% (2/30) ^D^
	2019	Puget Sound	Pt. Ludlow Hbr.	25 July–25 September	Cast Net	J	52 (2.4)–78 (6.8)	33% (20/60) ^D^
		Puget Sound	Pt. Angeles Hbr.	23 July–24 September	Cast Net	J	64 (6.7)–76 (7.0)	30% (18/60) ^D^
	2020	Puget Sound	Pt. Angeles Hbr.	23 June–20 October	Cast Net	J	37 (2.0)–63 (8.3)	63% (38/60) ^D^

“NA” indicates data “Not Available.” ^A^ Mass ranged between 2.2 and 3.6 g. ^B^ Photographs suggest 50–60 mm length. ^C^ Pacific herring *C. pallasii* were sampled from two locations in the north Puget Sound, WA region (San Juan Islands). External signs of VHS included hemorrhaging along the flank in a small proportion of individuals, increasing to approximately 10% throughout the summer. Samples from 2014 were not randomly collected; rather, individuals demonstrating external signs were selected, frozen at −20 °C, and submitted for laboratory diagnostics. ^D^ Results from Puget Sound locations in the period 2018–2020 reflect the peak infection prevalence from annual longitudinal sampling at each site. Results from all sampling dates are depicted in Figure 1A,B.

## Data Availability

The data that support the findings of this study are openly available at https://doi.org/10.5066/P9F55KEO (accessed on 12 July 2021).
